# A simulation study on missing data imputation for dichotomous variables using statistical and machine learning methods

**DOI:** 10.1038/s41598-023-36509-2

**Published:** 2023-06-09

**Authors:** Yingfeng Ge, Zhiwei Li, Jinxin Zhang

**Affiliations:** Department of Medical Statistics, School of Public Health, Sun Yat-Sen University, Guangzhou, 510080 People’s Republic of China

**Keywords:** Computational biology and bioinformatics, Scientific data, Statistics

## Abstract

The problem of missing data, particularly for dichotomous variables, is a common issue in medical research. However, few studies have focused on the imputation methods of dichotomous data and their performance, as well as the applicability of these imputation methods and the factors that may affect their performance. In the arrangement of application scenarios, different missing mechanisms, sample sizes, missing rates, the correlation between variables, value distributions, and the number of missing variables were considered. We used data simulation techniques to establish a variety of different compound scenarios for missing dichotomous variables and conducted real-data validation on two real-world medical datasets. We comprehensively compared the performance of eight imputation methods (mode, logistic regression (LogReg), multiple imputation (MI), decision tree (DT), random forest (RF), *k*-nearest neighbor (KNN), support vector machine (SVM), and artificial neural network (ANN)) in each scenario. Accuracy and mean absolute error (MAE) were applied to evaluating their performance. The results showed that missing mechanisms, value distributions and the correlation between variables were the main factors affecting the performance of imputation methods. Machine learning-based methods, especially SVM, ANN, and DT, achieved relatively high accuracy with stable performance and were of potential applicability. Researchers should explore the correlation between variables and their distribution pattern in advance and prioritize machine learning-based methods for practical applications when encountering dichotomous missing data.

## Introduction

Missing data is a common issue in medical research, and is often caused by human factors during data collection in epidemiology and clinical studies, which mainly involve individuals as subjects. Categorical data, particularly dichotomous variables, are commonly used in medical research. Dichotomous variables can only take two possible values, such as the presence or absence of a disease, benign or malignant pathology classification, and positive or negative test results. From the perspective of practical application, researchers usually discretize continuous variables into dichotomous or ordinal variables in the preprocessing period, to facilitate interpretation and assist decision-making.

Traditionally, there are three strategies for handling missing data, listwise deletion, weighting, and imputation. Further, the imputation methods can be divided into single imputation and multiple imputation. Single imputation, e.g., mean imputation, *k*-nearest neighbor imputation, regression imputation, and expectation maximization imputation, replace the missing value with a single estimated value and cannot reflect the uncertainty caused by missing data. Rubin^1^ proposed multiple imputation to overcome this shortcoming. In fact, as an imputation framework, multiple imputation can be embedded from parametric models such as RF, resampling method, and tree algorithm of iterative classification and regression to flexible Bayesian non-parametric model^2^. Moreover, multiple imputation based on the log-linear model^3^ and kernel function^4^ has also been proposed.

In addition to traditional statistical imputation methods, several machine learning techniques have also been applied to imputing missing data. Many researchers have successively used different datasets to compare the performance of traditional statistical and machine learning imputation methods, but the conclusions were different. Wei et al.^5^, Waljee et al.^6^, Shah et al.^7^ demonstrated respectively that RF outperforms other imputation methods in their datasets; Jerez et al.^8^, Zhou et al.^9^, Jadhav et al.^10^ found KNN outperforms other imputation methods in their datasets; Chlioui et al.^11^ found SVM performs best in two numeric datasets, while Tsai^12^ found DT performs best in mixed datasets. Furthermore, both the ensemble learning (EL) algorithm proposed by Wang^13^ and the generative adversarial imputation nets (GAIN) algorithm proposed by Dong^14^ have been reported as possessing satisfactory imputation performance.

Real datasets are often under specific missing mechanisms, correlation between variables, and value distributions. And extrapolating the results of the aforementioned simulation studies is a radical endeavor, given the limited simulated scenarios. Furthermore, the datasets used in these studies involve a wide range of variable types, and there remains a lack of research focusing on the factors that affect imputation performance and the most appropriate imputation methods for handling missing values in dichotomous data.

Considering different missing mechanisms, sample sizes, missing rates, the correlation between variables, value distributions, and the number of missing variables, our study constructed plenty of missing scenarios for dichotomous variables by simulation techniques. We comprehensively compared the performance of three traditional statistical methods (mode, LogReg, and MI) with five machine learning methods (DT, RF, KNN, SVM, and ANN) in each scenario to explore the stability and applicability of eight imputation methods.

## Methods

### Study design

**Figure 1:**
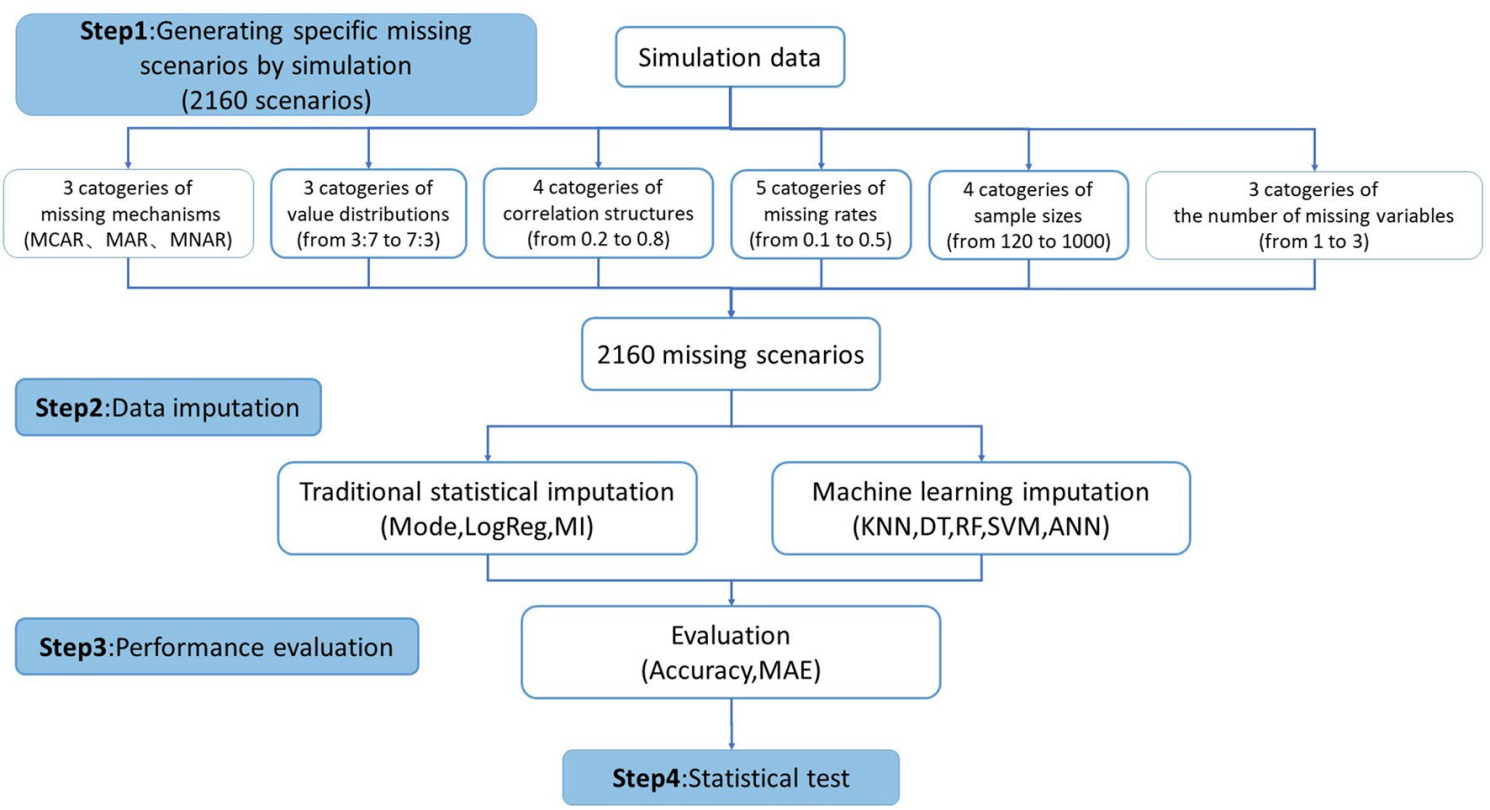
Study design.

The framework of the study design was shown in Fig. 1, which consisted of four main steps: generating specific missing scenarios by simulation, data imputation, performance evaluation, and statistical test.

### Generating specific missing scenarios by simulation

The pseudocode in Table 1 shows the basic flow of the simulation study. The simulation study considered multiple factors including missing mechanisms, sample sizes, missing rates, the correlation between variables, value distributions, and the number of missing variables. Rubin DB proposed three missing mechanisms including missing completely at random (MCAR, where the probability of missing data was independent of both observed and unobserved data), missing at random (MAR, where the probability of missing data was related to observed data but not to unobserved data), and missing not at random (MNAR, where the probability of missing data was related to both observed and unobserved data). Since the correlation between variables was considered in this study, the simulation process adopted the multivariate normal distribution as the basis. The datasets with dichotomous variables were generated by discretization. Then we established specific missing scenarios and imputation data. Value distribution was defined as the proportion of each classification that was discretized from the originally generated continuous variables following normal distribution into dichotomous variables, indicating the distribution of dichotomous variables. Value distribution was set with three patterns: 7:3, 5:5, and 3:7. Missing rate was applied to generating each missing variable, set into five circumstances: 10%, 20%, 30%, 40%, and 50%. The correlation structure was defined as the Pearson’s correlation coefficient, and four levels of 0.2, 0.4, 0.6, and 0.8 were used in this study. According to the study by Olivier^15^, it can be concluded that after being discretized from continuous variables following normal distribution to dichotomous variables, the contingency coefficient and the Pearson’s correlation coefficient were positively correlated when the sample size and marginal distribution were constant (both were set as constant in this study because the change in correlation coefficient was at the innermost level of the cycle). The sample size was set into four situations: 120, 200, 500, and 1000. The number of missing variables was set into three kinds: univariate missing, bivariate missing, and trivariate missing.

**Table 1:**
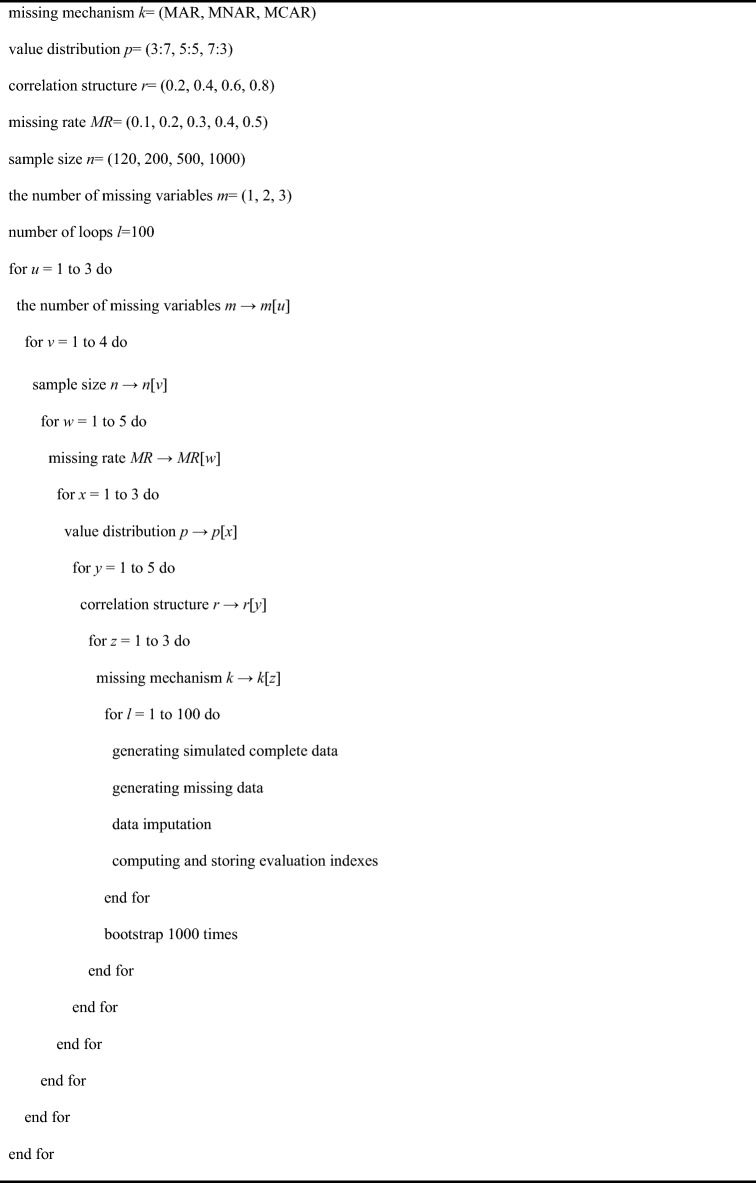
Simulation study pseudocode.

According to the study of Schouten et al.^16^, a multivariate normal distribution was generated, and the variables were denoted as *b*1*-b*7 respectively, to randomly generate dichotomous data where the mean vector was *μ* = (1, 1, 1, 1, 1, 1, 1), and the covariance matrix was

$$\sum =\left(\begin{array}{ccccccc}1& r& r& r& r& r& r\\ r& 1& r& r& r& r& r\\ r& r& 1& r& r& r& r\\ r& r& r& 1& r& r& r\\ r& r& r& r& 1& r& r\\ r& r& r& r& r& 1& r\\ r& r& r& r& r& r& 1\end{array}\right).$$

*r* was the Pearson’s correlation coefficient between variables, with the value of 0.2, 0.4, 0.6, and 0.8 respectively.

Taking trivariate missing as an example, the simulation process of trivariate missing mechanisms was as follows. MCAR: *b*1*–b*3 was randomly sampled without replacement, generating a specific missing rate. MAR: for the two value of *b*4 (0 and 1), two groups of random numbers were generated and sorted respectively. Then, *b*1 corresponding to the first *p*_*miss*_ × *n* random numbers in each group was set as missing: *p*_*miss*_ was the missing rate of 0 or 1, which can be calculated by sample size (*N*), missing rate, and the missing rate ratio of 0 and 1, which was 1:2; *n* was the number of each value. For example, if the sample size was 1000 and the dichotomous value distribution was 3:7, the corresponding n were 300 and 700 respectively. The calculation of missing values for b2 and b3 was similar, using the corresponding values of b5 and b6. MNAR: similar to the generation process of MAR, but the generation of missing values of *b*1–*b*3 was dependent on their own values. For the two values of *b*1 (0 or 1), two groups of random numbers were generated and sorted respectively. Then, the first *p*_*miss*_ × *n* random numbers of *b*1 in each group were set as missing. The calculation of missing values for b2 and b3 was again similar, using the corresponding values of b5 and b6.

Taking trivariate missing as an example, the whole simulation study was as follows. According to the sample size and correlation coefficient, the multivariate normal distribution matrix of *n* × 7 was simulated, and the variables were denoted as *b*1–*b*7. Sorted *b*1*–b*7 respectively, and discretized them into dichotomous values according to whether they were larger than the specific percentile (if the value distribution was 3:7, the corresponding percentile was P_30_). With *b*7 as the dependent variable and *b*1*–b*6 as the independent variables, a logistic regression model was accomplished to output the coefficients.

Considering the total missing rate, the missing scenarios of three mechanisms were generated based on the ratio of the missing rate of 0 and 1 which was set as 1:2, aiming to simulate the different probabilities of missing value in different circumstances. Finally, a total of 2160 scenarios with missing values were generated by simulation.

### Data imputation

This study shed light on the imputation performance comparison between three traditional statistical imputation methods (mode, LogReg, and MI) and five machine learning imputation methods (DT, RF, kNN, SVM, and ANN).

#### Traditional statistical methods

Mode imputation, one of the mean imputation methods, was applied to imputing categorical datasets and imputed the missing value with the mode of the variable containing the missing values.

LogReg imputation divided the original dataset into a complete dataset (D_com_) and an incomplete dataset (D_miss_) according to whether it contained missing values. Then, taking the missing variable *Y* as the dependent variable in D_com_ and selecting the appropriate independent variable *x*_i_ (i = 1,2,…,m), the binary logistic regression model was established to predict the missing value of the variable *Y* in D_miss_.

MI was proposed by Rubin DB and then improved by Meng and Schafer et al.^17,18^. MI originated from Bayesian statistics^19^, and the interpolation algorithm was used to impute the missing dataset for *m* times (generally *m* was no less than 5), and a complete dataset will be generated after each imputation, to obtain *m* complete datasets. Finally, the results of *m* imputation were summarized according to Rubin’s Rule^20^.

#### Machine learning methods

KNN imputation was an distance-based lazy classification method established based on the KNN algorithm proposed by Cover and Hart^21^. KNN imputation used the complete variables of the observations with missing values in the datasets to find out the *k* observations closest to them, and then used the mode of the corresponding values of these *k* observations as the imputation value of the missing values.

DT reflected the mapping relationship between object attributes and object values with a tree structure. The best feature and the best classification point will be selected each time as the decision or classification condition of the current node during construction, and the tree will be fully generated by classifying layer by layer until it cannot be divided or does not need to be divided^22^. We used the classification and regression tree (CART) algorithm to impute missing values in this study, and a CART model was built for the complete dataset to predict the corresponding missing values.

RF, derived from the random decision forest proposed by Tin^23^ and later developed by Breiman^24^, was an ensemble classifier composed of multiple decision tree models, whose final output classification was determined by the mode of all decision tree output categories. At first, *k* bootstrap subsample sets were randomly extracted from the complete original dataset, and a CART model was built for each subsample set to obtain *k* CART models. Finally, *k* models were voted on and summarized.

SVM, proposed by Vapnik et al.^25^, aiming to find an optimal hyperplane that can separate the two categories of data at maximum intervals. SVM imputation trained the model using the complete dataset, then applied the trained model to the missing dataset to predict the corresponding missing values.

ANN was a computational or mathematical model that mimicked the structure and function of the neural network of higher organisms (the central nervous system, especially the brain). The classic Back Propagation Neural Network (BPNN) was applied in this study, which was a multi-layer feedforward network with back propagation and error correction, including the input layer, hidden layer, and output layer. ANN imputation trained the network from the complete variables as network input, generating missing variables as output and training a most accurate network, then applied to the observation containing missing values.

### Performance evaluation

Accuracy was taken as the main performance indicator in this study which was shown in formula (1).

1 $$\mathrm{Accuracy }=\frac{{\mathrm{n}}_{\mathrm{cor}}}{{\mathrm{n}}_{\mathrm{imp}}},$$

where ${n}_{cor}$ is the amount of correct imputation for the variable under discussion and ${n}_{imp}$ is the amount of all imputation for the variable under discussion.

Also, as shown in formula (2), we used the MAE as a secondary indicator.

2 $$MAE=\frac{\sum_{i=1}^{n}\left|\widehat{{\beta }_{i}}-{\beta }_{i}\right|}{n},$$

where ${\beta }_{i}$ is the original coefficient and $\widehat{{\beta }_{i}}$ is the imputed coefficient.

In order to enhance the robustness, we conducted 1000 times bootstrap sampling for the results of every 100 repeated simulations in each missing scenario for all imputation methods, calculated the mean value and 95% confidence interval (percentile method) of two performance indicators, and took them as the final results in each scenario.

### Statistical test

To evaluate whether the observed performance differences between the eight methods under specific scenarios were statistically significant, the Kruskal–Wallis test was adopted in this study. We used multiple comparisons with the Bonferroni method to adjust the *P* values. The statistical significance level was 0.05.

We used R (version 4.0.0) to perform data simulation, statistical analysis, and plotting. The packages and main functions used in each imputation method were shown in Table 2. The computation was fulfilled in the high performance computer system in the School of Public Health, Sun Yat-Sen University, with the single-node 36-core setting.

**Table 2:** Packages and main functions required for the simulation study.

Methods	Packages	Functions
Mode	DMwR2	centralImputation()
LogReg	Base	glm()
MI	Mice	mice()
KNN	DMwR2	knnImputation()
DT	Rpart	rpart()
RF	missForest	missForest()
SVM	e1071	svm()
ANN	Nnet	nnet()

## Results

**Figure 2:**
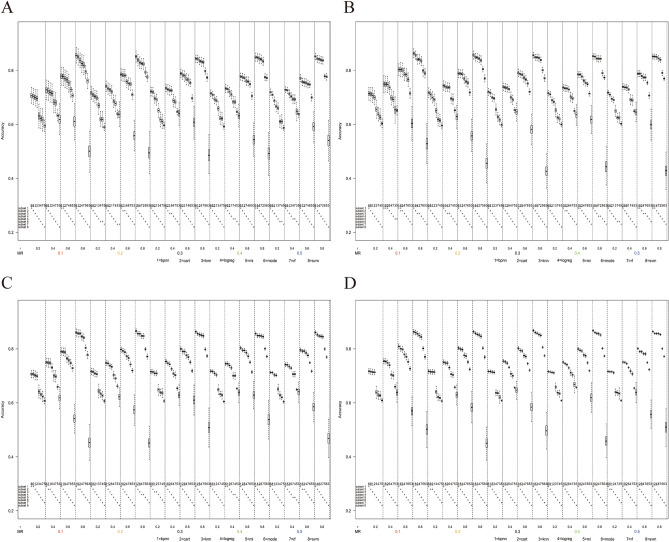
Box plots of accuracy in the scenario when the number of missing variables was one, the missing mechanism was MAR, the value distribution was 3:7. (**A**) The sample size n = 120. (**B**) The sample size n = 200. (**C**) The sample size n = 500. (D) The sample size n = 1000.

The results of this study were shown as Fig. 2, which depicted the profile that the accuracy or MAE of each imputation method with the change of missing rate (*MR*) and the Pearson’s correlation coefficient (*r*) under a specific scenario with a fixed number of missing variables, missing mechanism, value distribution, and sample size. Figure 2A corresponded to the scenario where the number of missing variables was one, the missing mechanism was MAR, the value distribution was 3:7, and the sample size was 120. The area between two vertical dashed grids represented a specific missing scenario, with the horizontal axis being double coordinates corresponding to a specific missing rate and correlation coefficient. The upper part of the area displayed box plots of the accuracy or MAE of each imputation method on bootstrap samples, while the lower part was the result of the pairwise comparison of the accuracy or MAE of eight imputation methods for each missing scenario. If the difference between two imputation methods was statistically significant, they belong to different subsets, with up to eight subsets for each scenario. The rank of each subset represented the relative performance of the eight imputation methods in that scenario, with subset 1 being the best-performing.

### Performance comparison with value distribution 3:7

#### Results in MAR mechanism

As shown in Fig. 2, under the MAR mechanism, the accuracy of each method had a small overall difference with the range being slightly more than 10% when the missing rate was 0.1 and the correlation coefficient was 0.2. With the increase of the correlation coefficient, the accuracy difference gradually increased to nearly 40%. SVM, ANN, and DT showed high accuracy and stability in all scenarios with the lowest accuracy being higher than 70%. With the increase of the correlation coefficient, the imputation accuracy of these three methods increased rapidly. The accuracies of LogReg, RF, and MI also increased with the increase of the correlation coefficient but were inferior to the above three methods. When the correlation coefficient was 0.2, the accuracy of mode imputation was the highest among the eight methods, but with the growth of the correlation coefficient, its growth rate was the slowest, resulting in declining of the relative ranks when the correlation coefficient was high. It was noteworthy that the accuracy of KNN decreased with the increase of the correlation coefficient, and the accuracy of KNN was at the lower level.

**Figure 3:**
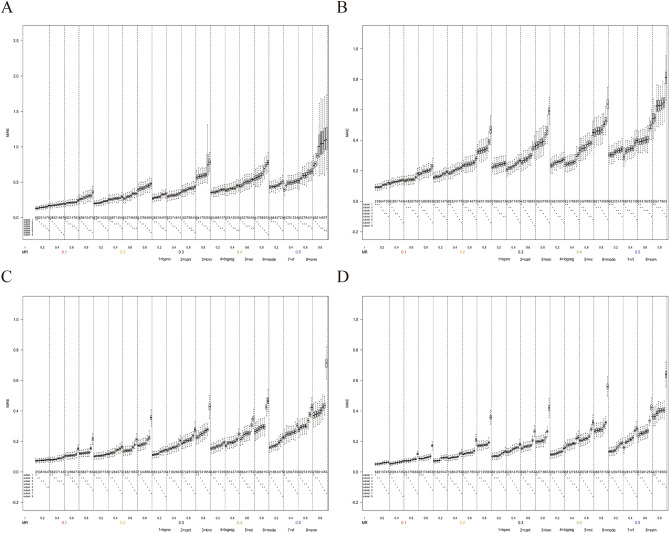
Box plots of MAE in the scenario when the number of missing variables was one, the missing mechanism was MAR, the value distribution was 3:7. (**A**) The sample size n = 120. (**B**) The sample size n = 200. (**C**) The sample size n = 500. (**D**) The sample size n = 1000.

Figure 3 shows that under the MAR mechanism, the results of MAE varied with missing rate and the correlation coefficient when the number of missing variables was one, the value distribution was 3:7, and the sample sizes were 120, 200, 500, and 1000, respectively. The results showed that the MAE of KNN was significantly greater than that of other methods only in the scenario of the high correlation coefficient. However, the difference between MAE among other methods was not significant, and there were no obvious superiority or inferiority. Since MAE was a secondary evaluation indicator in this study and the regularity was similar to the results in this section in most scenarios, subsequent relevant results about MAE were included in the Supplement 1.

The changes in the missing rate and sample size merely affected the accuracy of each method, but the increase in the missing rate will slightly reduce the imputation accuracy and increase the values of MAE, and the increase in sample size had little effect on the imputation accuracy but will slightly increase the stability of MAE (decrease of dispersion according to the box plot). In other scenarios in our study, similar results could be produced when sample size was changed but other parameters were fixed. Therefore, the following scenarios only showed the results when the sample size was 1000, and refer to Supplement 2 for the results of other sample sizes. When the missing variable increased from one to three, the rank of accuracy of each method kept almost stable, but the accuracy with KNN was slightly increased, so the overall range was narrow on the whole, but the rank remained approximately same. The MAE stability of each method increased, and the overall range increased slightly. Meanwhile, the MAE with RF increased significantly, that is, the relative rank decreased (refer to Supplement 1 and Supplement 2 for the MAE and accuracy results respectively with different numbers of missing variables).

#### Results in MNAR mechanism

**Figure 4:**
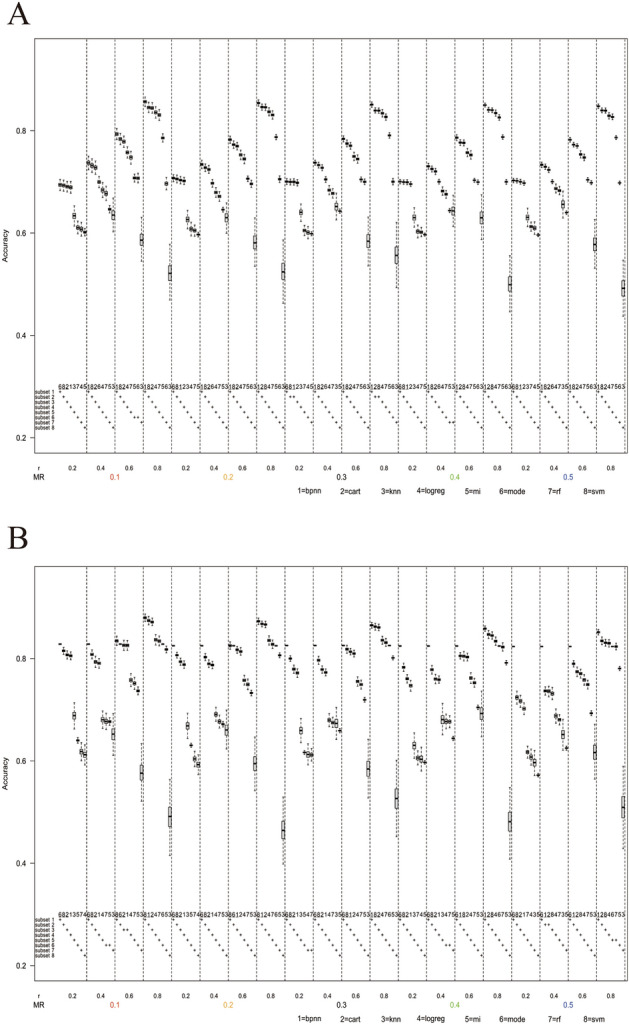
Box plots of accuracy in the scenario when the number of missing variables was one, the value distribution was 3:7, the sample size n = 1000. (**A**) The missing mechanism was MNAR. (**B**) The missing mechanism was MCAR.

As shown in Fig. 4, under the MNAR mechanism, the accuracy of each method had a small overall difference with the range being nearly 20% when the missing rate was 0.1 and the correlation coefficient was 0.2. With the increase of the correlation coefficient, the accuracy difference gradually increased to nearly 40%. Similarly, SVM, ANN, and DT were superior to other methods with high accuracy in any scenario.

#### Results in MCAR mechanism

As shown in Fig. 4, under the MCAR mechanism, the accuracy of each method had a small overall difference with the range being slightly more than 10% when the missing rate was 0.1 and the correlation coefficient was 0.2. With the increase of the correlation coefficient, the accuracy difference gradually increased to nearly 35%. Mode, SVM, ANN, and DT were superior to other methods with high accuracy. The accuracy of mode imputation remained a stable level when the correlation coefficient was increased, while the accuracies of the other three methods all increased and were very close when the correlation coefficient was high.

### Performance comparison with value distribution 7:3

#### Results in MAR mechanism

**Figure 5:**
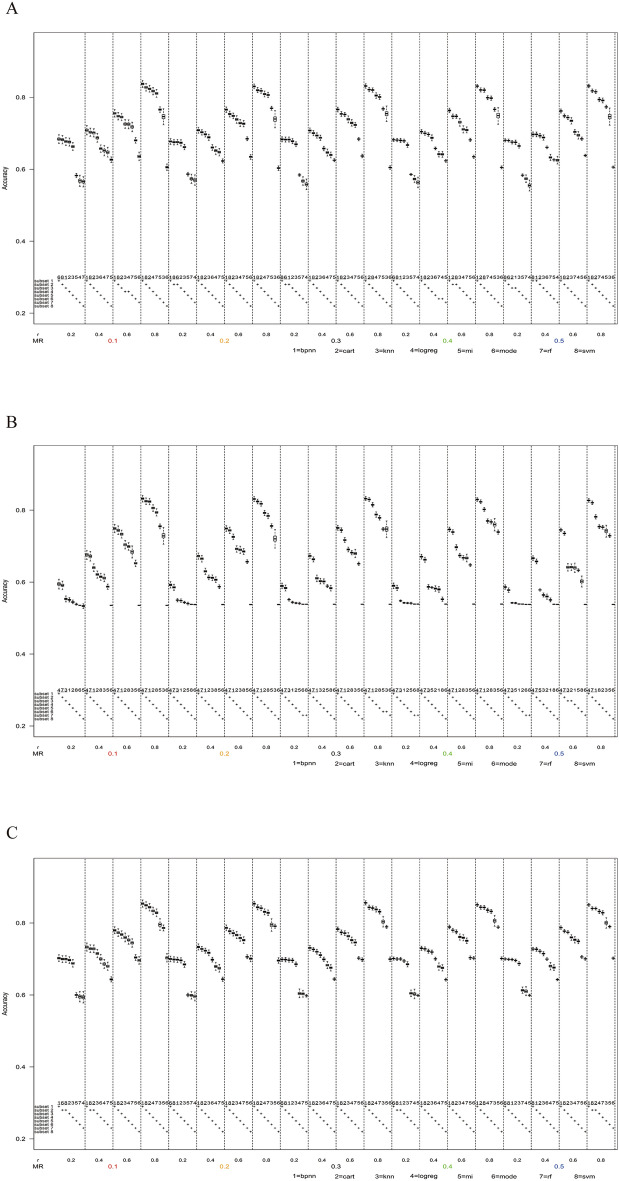
Box plots of accuracy in the scenario when the number of missing variables was one, the value distribution was 7:3, the sample size n = 1000. (**A**) The missing mechanism was MAR. (**B**) The missing mechanism was MNAR. (**C**) The missing mechanism was MCAR.

As shown in Fig. 5, under the MAR mechanism, the accuracy of each method had a small overall difference with the range being slightly wider than 10% when the missing rate was 0.1 and the correlation coefficient was 0.2. With the increase of the correlation coefficient, the accuracy difference gradually increased to nearly 30%. SVM, ANN, and DT were still the three most stable methods. It was worth noting that accuracy using KNN increased slightly with the increase of correlation coefficient, while accuracy using mode decreased slightly with the increase of correlation coefficient.

#### Results in MNAR mechanism

As shown in Fig. 5, under the MNAR mechanism, the accuracy of each method had a small overall difference with the range being slightly narrower than 10% when the missing rate was 0.1 and the correlation coefficient was 0.2. With the increase of the correlation coefficient, the accuracy difference gradually increased to nearly 30%. Under MNAR mechanism, the performance of all imputation methods was not ideal when the correlation coefficient was 0.2. It was noteworthy that LogReg and RF had the best accuracies, but because of the small overall range among the eight methods, the performance of SVM, ANN, and DT were still stable.

#### Results in MCAR mechanism

As shown in Fig. 5, under the MCAR mechanism, the accuracy of each method had a small overall difference with the range being slightly wider than 10% when the missing rate was 0.1 and the correlation coefficient was 0.2. With the increase of the correlation coefficient, the accuracy difference slightly increased to nearly 15%. SVM, ANN, and DT outperformed the other methods, and the difference between KNN and the above three methods was small.

### Performance comparison with value distribution 5:5

#### Results in MAR mechanism

**Figure 6:**
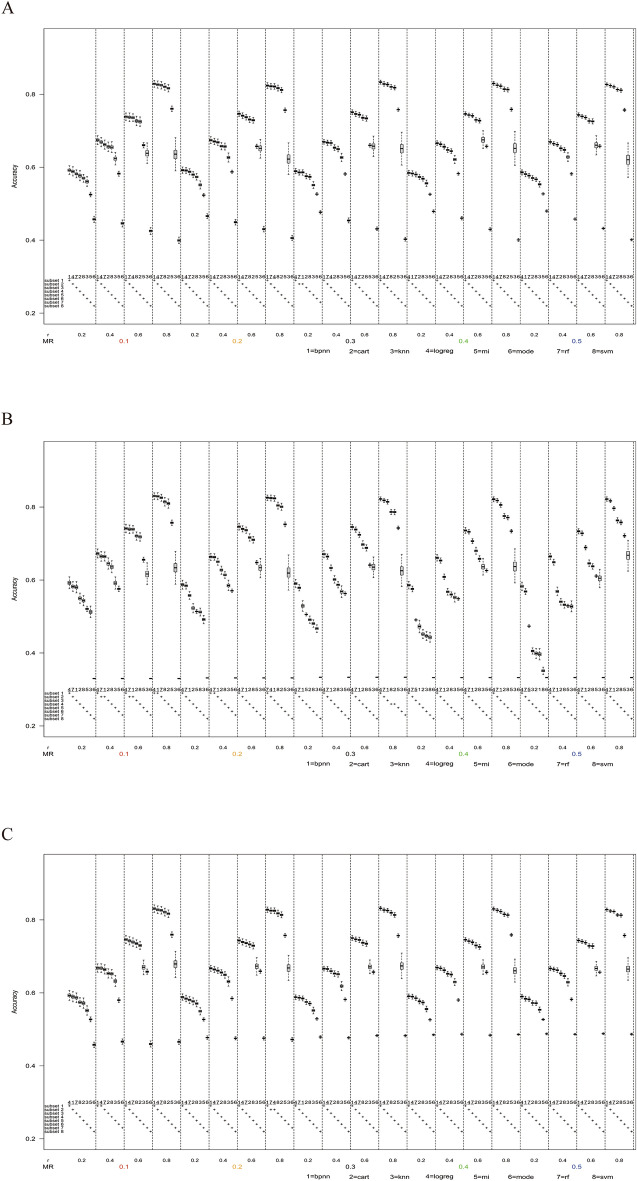
Box plots of accuracy in the scenario when the number of missing variables was one, the value distribution was 5:5, the sample size n = 1000. (**A**) The missing mechanism was MAR. (**B**) The missing mechanism was MNAR. (**C**) The missing mechanism was MCAR.

As shown in Fig. 6, under the MAR mechanism, ANN, LogReg, RF, DT, and SVM showed high accuracy with stability, ranking in the top five in all scenarios with a smaller difference. The accuracy of KNN and MI also increased with the increase of the correlation coefficient, but the increasing rate was slightly lower than that of the above five methods. It was worth noting that the accuracy of the mode decreased with the increase of the correlation coefficient, and its accuracy was significantly lower than that of other methods.

#### Results in MNAR mechanism

As shown in Fig. 6, under the MNAR mechanism, the performance of all imputation methods was not ideal when the correlation coefficient was 0.2. The accuracy of LogReg and RF was slightly higher than that of ANN, and the difference was small except for mode, with an accuracy of less than 0.6.

#### Results in MCAR mechanism

As shown in Fig. 6, under the MCAR mechanism, the accuracy among the seven methods had a small overall difference except for mode imputation. ANN, LogReg, RF, and SVM showed high accuracy with stability, ranking in the top four in all scenarios with a smaller difference. The accuracy of the mode was stable between 40% and 50%, ranked as the worst one of all methods.

As shown in Table 3, we concluded a few of recommended imputation methods in above scenarios.

**Table 3:** A few of recommended imputation methods in specific scenarios.

Value distribution	Missing mechanism	Correlation structure	Recommended methods							
			Mode	LogReg	MI	DT	RF	KNN	SVM	ANN
Negative event < positive event (e.g. 3:7)	MAR	< 0.5	***	*	–	***	*	–	*	***
		> 0.5	–	*	–	***	*	–	***	***
	MNAR	< 0.5	***	–	–	***	–	–	***	*
		> 0.5	***	–	–	*	–	–	***	***
	MCAR	< 0.5	*	–	–	*	–	–	***	***
		> 0.5	*	*	–	*	*	–	***	***
Negative event ≈positive event (e.g. 5:5)	MAR	< 0.5	–	***	–	*	***	–	*	***
		> 0.5	–	***	–	*	***	–	*	***
	MNAR	< 0.5	–	*	–	–	*	–	–	*
		> 0.5	–	***	–	*	***	–	*	***
	MCAR	< 0.5	–	***	–	*	***	–	***	***
		> 0.5	–	***	–	*	***	–	*	***
Negative event > positive event (e.g. 7:3)	MAR	< 0.5	***	–	–	*	–	*	***	***
		> 0.5	–	–	–	***	*	*	***	***
	MNAR	< 0.5	–	*	–	*	*	*	–	*
		> 0.5	–	*	–	*	*	–	*	*
	MCAR	< 0.5	*	–	–	***	–	*	***	***
		> 0.5	–	*	–	***	*	*	***	***

***Recommended methods.

*Acceptable methods.

– Not recommended methods.

## Discussion

Aiming to impute missing dichotomous data in medical research, we found machine learning imputation methods outperformed traditional statistical imputation methods. The methods based on machine learning techniques, especially SVM, ANN, and DT, can achieve relatively high accuracy with stable performance and wide applicability. This simulation study demonstrated that missing mechanisms, value distributions, and the correlation between variables were the main factors affecting the relative performance of imputation methods. However, sample sizes, missing rates, and the number of missing variables were not the main factors affecting the relative performance of the eight imputation methods and had little influence on the performance of each imputation method.

SVM and ANN can solve prediction issues in both linear and nonlinear classification, resulting in outstanding performance in most simulation scenarios. In this study, we utilized DT with the CART algorithm, which demonstrated high accuracy and stable performance due to the absence of marked non-homogeneous characteristics in the random simulation data. Relevant studies^11,12,26^ also demonstrated that the above three imputation methods had excellent performance when applied to datasets with continuous and mixed variables. Our study supported these findings from the perspective of missing dichotomous variables.

KNN and RF have been reported to have excellent imputation performance in relevant studies^5–10,27^, but these researches were based on real-data applications with continuous or mixed variables in limited application scenarios. In this study, KNN only had a relatively moderate imputation accuracy in scenarios with a value distribution of 7:3, but it had poor performance in most scenarios, which may be caused by the local structure of data in these simulation scenarios. On the other hand, when the data distribution was balanced (value distribution of 5:5), RF had relatively high accuracy. However, in the scenarios of other value distributions, RF had relatively low accuracy. This was because bootstrap sampling process wasn’t prone to generate bias under the balanced data, but it was not easy to achieve in the case of other value distributions. Our study showed that KNN and RF, which had outstanding imputation performance in missing continuous data, may not reach similar performance in specific missing dichotomous data, especially non-normality and imbalanced data. This finding was consistent with relevant studies^14,27^. It suggested the specific data missing situation should be examined carefully in practical application.

Mode imputation merely considered the correlation between variables, though the imputation accuracy was stable in most missing scenarios. However, with the increase of correlation coefficient, the relative performance will be inevitably declined. We observed LogReg and MI had lower imputation accuracy when the correlation coefficient was 0.2. With the increase of the correlation coefficient, the accuracy increased, which was consistent with the research results reported by Zhang^28^. In this study, the logistic algorithm was also used for MI, so both of them needed to meet the condition of linear or approximate linear correlation between missing variables and imputation variables. Therefore, these two imputation methods were inevitably subject to the same constraints, which made them less outstanding in performance evaluation. Therefore, it was questionable to use LogReg and MI to deal with missing values subjectively in common medical research.

We choose accuracy as the main evaluation indicator in this study, with MAE used as a secondary exploratory evaluation indicator to reflect the relationship between missing variables and dependent variables. The simulation results showed that in most missing scenarios, there was no marked difference in MAE among the various methods, which was consistent with the results reported by Tsai^12^ and Guo^29^ using root mean square error (RMSE) as an indicator. It was indicated that in most scenarios, the fitness levels were approximately equal after imputation was performed using the eight methods, reflecting the robustness of various methods in exploring regression relationships in this study. Mode imputation replaced missing values with mode, leading to smaller variance and smaller MAE when data were unbalanced distributed, suggesting that value distribution affected imputation performance. SVM, ANN, and DT exhibited high accuracy and low MAE, making them the methods with superior and comprehensive performance.

This study focused on evaluating the imputation performance of dichotomous variables. Comprehensively considering the influence of various factors on the imputation performance of the selected methods, we generated specific missing scenarios by simulation, and used the bootstrap method for robust estimation of eight imputation methods, to compare imputation performance using indicators of accuracy and MAE. Also, the results of two real-world datasets were consistent with the results of simulated research under corresponding scenarios (refer to Supplement 3). However, our study also has limitations. Firstly, we only conducted comprehensive but idealized simulation experiments and validated on limited real-world datasets in this study, and we will examine more real-data applications to verify the conclusions of this simulation study. Secondly, the simulation comparison framework of this study was based on dichotomous variables, and further research could focus on the dataset containing continuous, ordinal, or nominal variables. Thirdly, we choose eight imputation methods based on practical considerations, so default parameters among the eight methods were applied in this study. In the future, we will further explore the improved methods or other imputation methods to impute missing data.

## Conclusions

We demonstrated that machine learning-based methods, especially SVM, ANN, and DT, can achieve relatively high accuracy with stable performance and wide applicability, and researchers can prioritize them for practical applications. This simulation study showed that missing mechanisms, value distributions and correlation between variables were the main factors affecting the relative performance of imputation methods. Researchers should explore the value distributions and correlation between variables in advance and prioritize machine learning-based methods for practical applications when encountering dichotomous missing data.

## Supplementary Information

Supplementary Information 1.

Supplementary Information 2.

Supplementary Information 3.

Supplementary Information 4.

## Data Availability

Please refer to Supplement 4 for the partial codes of simulated study. The complete codes and datasets used during the current study are available from the corresponding author on reasonable request.

## Abbreviations

ANN: Artificial neural network
BPNN: Back propagation neural network
CART: Classification and regression tree
DT: Decision tree
EL: Ensemble learning
KNN: *k*-Nearest neighbor
GAIN: Generative adversarial imputation nets
LogReg: Logistic regression
MAE: Mean absolute error
MAR: Missing at random
MCAR: Missing completely at random
MI: Multiple imputation
MNAR: Missing not at random
RF: Random forest
RMSE: Root mean square error
SVM: Support vector machine
