# Supplementary material for: A simulation study on missing data imputation for dichotomous variables using statistical and machine learning methods
Source: Sci Rep. 2023 Jun 9;13:9432. doi: 10.1038/s41598-023-36509-2 (PMC10256703; doi:10.1038/s41598-023-36509-2)
Supplement: Supplementary file 3 — Supplementary Information 3. [file 41598_2023_36509_MOESM3_ESM.zip › Real data validation.docx]

Real-data validation

This study analyzed survival data from 382 drug-resistant tuberculosis patients from a hospital in Guangzhou City, China and 1030 hospitalized patients in the infectious disease department from a hospital in Shanxi Province, China. Taking into account the potential occurrence of MAR and MCAR mechanisms in real-world data, missing data simulation will be conducted on two real data. Accuracy and mean absolute error (MAE) were applied to evaluating the consistency between the results of real-data analysis and the simulation studies.

**MCAR in real data**

Using drug-resistant tuberculosis dataset, with the presence or absence of diabetes as the missing variable, MCAR mechanism was created with missing rates of 10%, 20%, 30%, 40%, and 50%. The simulation was repeated 100 times for each missing rate, and the average accuracy after imputation was calculated. Furthermore, considering that various forms of loss to follow-up due to diabetes were common in actual situations, which was an important influencing factor in the treatment of drug-resistant tuberculosis, a regression relationship was constructed with the occurrence of adverse treatment outcomes as the dependent variable and the presence or absence of diabetes and three imputed variables as independent variables. The MAE was calculated using regression relationship.

**Table 1** Value distribution of drug-resistant tuberculosis dataset (n=382)

| Variable (value) | n | P (%) |
| --- | --- | --- |
| Diabetes |  |  |
| absence（0） | 261 | 68.3 |
| presence（1） | 121 | 31.7 |
| Drinking |  |  |
| absence（0） | 350 | 91.6 |
| presence（1） | 32 | 8.4 |
| Smoking |  |  |
| absence（0） | 284 | 74.3 |
| presence（1） | 98 | 25.7 |
| Hypertension |  |  |
| absence（0） | 367 | 96.0 |
| presence（1） | 15 | 4.0 |
| Gender |  |  |
| female（0） | 111 | 29.1 |
| male（1） | 271 | 70.9 |
| Age |  |  |
| ≤45（0） | 175 | 45.8 |
| ＞ 45（1） | 207 | 54.2 |
| Household registration |  |  |
| within Guangzhou City（0） | 199 | 52.1 |
| outside Guangzhou City（1） | 183 | 47.9 |
| Pulmonary tuberculosis cavities |  |  |
| absence（0） | 151 | 39.5 |
| presence（1） | 231 | 60.5 |
| TEAE |  |  |
| absence（0） | 279 | 73.0 |
| presence（1） | 103 | 27.0 |

Note: TEAE: treatment emergent adverse event.

**Table 2** Correlation coefficients between each variable and diabetes

| Variable | Correlation coefficients |
| --- | --- |
| Drinking | 0.118 |
| Smoking | 0.127 |
| Hypertension | 0.150 |
| Gender | 0.208 |
| Age | 0.362 |
| Household registration | 0.198 |
| Pulmonary tuberculosis cavities | 0.179 |

Note: The underscore represents the selected imputed variables.

**Table 3** Accuracy for the drug-resistant tuberculosis dataset

| Missing Rate | Mode | LogReg | MI | KNN | DT | RF | SVM | ANN |
| --- | --- | --- | --- | --- | --- | --- | --- | --- |
| 10% | 0.691  (0.677,0.706) | 0.644  (0.630,0.657) | 0.580  (0.565,0.595) | 0.685  (0.668,0.701) | 0.686  (0.669,0.702) | 0.642  (0.629,0.656) | 0.686  (0.672,0.702) | 0.683  (0.667,0.691) |
| 20% | 0.688  (0.678,0.697) | 0.645  (0.636,0.655) | 0.572  (0.561,0.584) | 0.681  (0.671,0.690) | 0.681  (0.673,0.690) | 0.643  (0.634,0.653) | 0.679  (0.670,0.688) | 0.683  (0.675,0.691) |
| 30% | 0.684  (0.678,0.690) | 0.640  (0.633,0.647) | 0.576  (0.567,0.585) | 0.677  (0.670,0.685) | 0.679  (0.673,0.686) | 0.640  (0.633,0.647) | 0.677  (0.671,0.684) | 0.683  (0.677,0.689) |
| 40% | 0.683  (0.677,0.688) | 0.637  (0.631,0.642) | 0.579  (0.571,0.588) | 0.674  (0.668,0.679) | 0.679  (0.673,0.684) | 0.637  (0.632,0.643) | 0.676  (0.671,0.682) | 0.681  (0.676,0.686) |
| 50% | 0.685  (0.681,0.689) | 0.644  (0.638,0.649) | 0.579  (0.572,0.585) | 0.678  (0.673,0.682) | 0.679  (0.674,0.684) | 0.643  (0.638,0.649) | 0.680  (0.676,0.684) | 0.685  (0.680,0.690) |

Note: mean (lower limits, upper limits), the underscore represents that the method has a higher ranking in terms of accuracy.

**Table 4** MAE for the drug-resistant tuberculosis dataset

| Missing Rate | Mode | LogReg | MI | KNN | DT | DT | SVM | ANN |
| --- | --- | --- | --- | --- | --- | --- | --- | --- |
| 10% | 0.079  (0.069,0.091) | 0.081  (0.070,0.093) | 0.077  (0.067,0.088) | 0.086  (0.076,0.096) | 0.080  (0.069,0.091) | 0.082  (0.076,0.096) | 0.080  (0.069,0.091) | 0.080  (0.070,0.091) |
| 20% | 0.108  (0.093,0.125) | 0.114  (0.099,0.130) | 0.126  (0.109,0.141) | 0.121  (0.106,0.136) | 0.114  (0.099,0.130) | 0.113  (0.098,0.128) | 0.113  (0.099,0.128) | 0.108  (0.094,0.123) |
| 30% | 0.146  (0.126,0.168) | 0.151  (0.131,0.175) | 0.177  (0.157,0.196) | 0.149  (0.129,0.170) | 0.142  (0.124,0.161) | 0.154  (0.131,0.176) | 0.150  (0.129,0.171) | 0.144  (0.123,0.164) |
| 40% | 0.180  (0.153,0.208) | 0.199  (0.171,0.228) | 0.222  (0.193,0.249) | 0.185  (0.157,0.215) | 0.172  (0.148,0.201) | 0.199  (0.173,0.230) | 0.180  (0.154,0.209) | 0.185  (0.160,0.211) |
| 50% | 0.200  (0.169,0.233) | 0.210  (0.177,0.242) | 0.294  (0.268,0.323) | 0.209  (0.180,0.240) | 0.211  (0.180,0.243) | 0.214  (0.185,0.246) | 0.212  (0.182,0.241) | 0.208  (0.181,0.239) |

Note: mean (lower limits, upper limits).

The value distribution in the drug-resistant tuberculosis dataset and the correlation coefficients between each variable and diabetes can be found in Table 1 and Table 2, respectively. In this case, the data closely resemble the conditions of a simulation experiment with univariate missing, MCAR mechanism, a sample size of 500, a value distribution of 7:3, and a correlation coefficient of 0.2. In this example, whether the individual drinks alcohol, smokes, and has hypertension were selected as the imputed variables (indicated by an underscore in Table 2). The results in Table 3 demonstrate that the imputation accuracy in the real-data followed a similar pattern to the corresponding scenario in the simulation study. Specifically, Mode, SVM, ANN, DT, and KNN were relatively better (indicated by an underscore in Table 3), while MI, RF, and LogReg were relatively poorer methods. Additionally, the results in Table 4 shows that as the missing rate increased, there was an increasing tendency for the MAE. The differences in MAE among different methods were not significant for the same missing rate, which was consistent with the corresponding scenario in the simulation study.

**MAR in real data**

We utilized an infectious disease dataset, where the missingness of the history of cerebrovascular disease variable was contingent upon the presence or absence of a history of cardiovascular disease. MAR mechanism was created with missing rates of 10%, 20%, 30%, 40%, and 50%. The simulation was repeated 100 times for each missing rate, and the average accuracy after imputation was calculated. A regression relationship was constructed with the patient's cure status as the dependent variable and the presence or absence of a history of cerebrovascular disease and imputed variables as independent variables. The MAE was calculated using regression relationship.

**Table 5** Value distribution of infectious disease dataset (n=1030)

| Variable (value) | n | P (%) |
| --- | --- | --- |
| History of cerebrovascular disease |  |  |
| absence（0） | 734 | 71.3 |
| presence（1） | 296 | 28.7 |
| History of cardiovascular disease |  |  |
| absence（0） | 642 | 62.3 |
| presence（1） | 388 | 37.7 |
| Myocardial infarction |  |  |
| absence（0） | 967 | 93.9 |
| presence（1） | 63 | 6.1 |
| Peripheral vascular disease |  |  |
| absence（0） | 925 | 89.8 |
| presence（1） | 105 | 10.2 |
| Diabetes |  |  |
| female（0） | 858 | 83.3 |
| male（1） | 172 | 16.7 |
| Hemiplegia |  |  |
| absence（0） | 1017 | 98.7 |
| presence（1） | 13 | 0.3 |
| Gastrointestinal disorders |  |  |
| absence（0） | 918 | 89.1 |
| presence（1） | 112 | 10.9 |
| COPD |  |  |
| absence（0） | 941 | 91.4 |
| presence（1） | 89 | 8.6 |
| AIDS |  |  |
| absence（0） | 1030 | 100.0 |
| presence（1） | 0 | 0.0 |
| Age |  |  |
| ≤45（0） | 208 | 20.2 |
| >45（1） | 822 | 79.8 |
| Cure |  |  |
| absence（0） | 907 | 88.1 |
| presence（1） | 123 | 11.9 |

Note: COPD: chronic obstructive pulmonary disease; AIDS: Acquired immunodeficiency syndrome.

**Table 6** Correlation coefficients between each variable and the history of cerebrovascular disease

| Variable | Correlation coefficients |
| --- | --- |
| History of cardiovascular disease | 0.189 |
| Myocardial infarction | 0.079 |
| Peripheral vascular disease | 0.069 |
| Diabetes | 0.100 |
| Hemiplegia | 0.014 |
| Gastrointestinal disorders | 0.008 |
| COPD | 0.004 |
| AIDS | - |
| Age | 0.100 |

Note: 1. “-” indicates that the correlation coefficient is not calculable;

2. The underscore represents the selected imputed variables.

3. COPD: chronic obstructive pulmonary disease; AIDS: Acquired immunodeficiency syndrome.

**Table 7** Accuracy for the infectious disease dataset

| Missing Rate | Mode | LogReg | MI | KNN | DT | RF | SVM | ANN |
| --- | --- | --- | --- | --- | --- | --- | --- | --- |
| 10% | 0.681  (0.674,0.688) | 0.575  (0.565,0.584) | 0.571  (0.563,0.580) | 0.653  (0.641,0.664) | 0.681  (0.674,0.688) | 0.574  (0.565,0.585) | 0.681  (0.674,0.688) | 0.681  (0.674,0.688) |
| 20% | 0.682  (0.677,0.687) | 0.571  (0.565,0.578) | 0.574  (0.569,0.580) | 0.651  (0.639,0.659) | 0.681  (0.677,0.686) | 0.572  (0.565,0.578) | 0.681  (0.677,0.686) | 0.681  (0.677,0.686) |
| 30% | 0.682  (0.678,0.686) | 0.569  (0.562,0.576) | 0.576  (0.570,0.581) | 0.647  (0.635,0.658) | 0.679  (0.674,0.683) | 0.576  (0.570,0.581) | 0.680  (0.677,0.684) | 0.678  (0.675,0.682) |
| 40% | 0.681  (0.679,0.684) | 0.565  (0.558,0.572) | 0.579  (0.574,0.584) | 0.659  (0.651,0.665) | 0.675  (0.672,0.678) | 0.573  (0.567,0.578) | 0.680  (0.677,0.682) | 0.673  (0.670,0.677) |
| 50% | 0.682  (0.679,0.684) | 0.570  (0.563,0.576) | 0.579  (0.575,0.583) | 0.665  (0.662,0.668) | 0.674  (0.671,0.677) | 0.572  (0.565,0.579) | 0.679  (0.676,0.681) | 0.670  (0.666,0.673) |

Note: mean (lower limits, upper limits), the underscore represents that the method has a higher ranking in terms of accuracy.

**Table 8** MAE for infectious disease dataset

| Missing Rate | Mode | LogReg | MI | KNN | DT | RF | SVM | ANN |
| --- | --- | --- | --- | --- | --- | --- | --- | --- |
| 10% | 0.069  (0.061,0.079) | 0.083  (0.071,0.096) | 0.070  (0.059,0.081) | 0.070  (0.061,0.079) | 0.069  (0.061,0.079) | 0.085  (0.073,0.096) | 0.069  (0.061,0.078) | 0.069  (0.060,0.079) |
| 20% | 0.111  (0.094,0.131) | 0.127  (0.110,0.146) | 0.117  (0.101,0.133) | 0.118  (0.098,0.138) | 0.112  (0.094,0.130) | 0.128  (0.110,0.146) | 0.110  (0.091,0.128) | 0.109  (0.092,0.126) |
| 30% | 0.135  (0.115,0.159) | 0.156  (0.136,0.178) | 0.144  (0.124,0.163) | 0.139  (0.119,0.159) | 0.138  (0.118,0.158) | 0.153  (0.133,0.175) | 0.134  (0.114,0.155) | 0.139  (0.118,0.162) |
| 40% | 0.164  (0.141,0.190) | 0.175  (0.149,0.201) | 0.173  (0.152,0.196) | 0.176  (0.149,0.206) | 0.166  (0.141,0.193) | 0.165  (0.141,0.189) | 0.165  (0.140,0.191) | 0.177  (0.151,0.204) |
| 50% | 0.189  (0.158,0.230) | 0.169  (0.144,0.195) | 0.192  (0.170,0.213) | 0.187  (0.157,0.218) | 0.187  (0.158,0.227) | 0.160  (0.136,0.187) | 0.193  (0.161,0.232) | 0.185  (0.161,0.213) |

Note: mean (lower limits, upper limits).

The value distribution in the infectious disease dataset and the correlation coefficients between each variable and the history of cerebrovascular disease can be found in Table 5 and Table 6, respectively. In this case, the data closely resemble the conditions of a simulation experiment with univariate missing, MAR mechanism, a sample size of 1000, a value distribution of 7:3, and a correlation coefficient of 0.2. In this example, age greater than 45 years, presence or absence of myocardial infarction, presence or absence of peripheral vascular disease, and presence or absence of diabetes were selected as imputed variables (indicated by an underscore in Table 6). The results in Table 7 demonstrate that the imputation accuracy in the real-data followed a similar pattern to the corresponding scenario in the simulation study. Specifically, Mode, SVM, ANN, DT, and KNN were relatively better (indicated by an underscore in Table 7), while MI, RF, and LogReg were relatively poorer methods. Additionally, the results in Table 8 shows that as the missing rate increased, there was an increasing tendency for the MAE. The differences in MAE among different methods were not significant for the same missing rate, which was consistent with the corresponding scenario in the simulation study.
